# Regional-level risk factors for severe hand-foot-and-mouth disease: an ecological study from mainland China

**DOI:** 10.1186/s12199-020-00927-9

**Published:** 2021-01-08

**Authors:** Qing Pan, Fengfeng Liu, Juying Zhang, Xing Zhao, Yifan Hu, Chaonan Fan, Fan Yang, Zhaorui Chang, Xiong Xiao

**Affiliations:** 1 Department of Epidemiology and Biostatistics, West China School of Public Health and West China Fourth Hospital, Sichuan University, Chengdu, Sichuan 610041 PR China; 2 Division of Infectious Disease & Key Laboratory of Surveillance and Early Warning on Infectious Disease, Chinese Center for Disease Control and Prevention, Beijing, 102206 PR China

**Keywords:** Hand, foot and mouth disease, Case-severity rate, City-specific characteristics, Ecological study

## Abstract

**Background:**

Severe hand-foot-and-mouth disease (HFMD) is a life-threatening contagious disease among young children and infants. Although enterovirus A71 has been well acknowledged to be the dominant cause of severe HFMD, there still remain other unidentified risk factors for severe HFMD. Previous studies mainly focused on identifying the individual-level risk factors from a clinical perspective, while rare studies aimed to clarify the association between regional-level risk factors and severe HFMD, which may be more important from a public health perspective.

**Methods:**

We retrieved the clinical HFMD counts between 2008 and 2014 from the Chinese Center for Disease Control and Prevention, which were used to calculated the case-severity rate in 143 prefectural-level cities in mainland China. For each of those 143 cities, we further obtained city-specific characteristics from the China City Statistical Yearbook (social and economic variables) and the national meteorological monitoring system (meteorological variables). A Poisson regression model was then used to estimate the associations between city-specific characteristics (reduced by the principal component analysis to avoid multicollinearity) and the case-severity rate of HFMD. The above analysis was further stratified by age and gender to examine potential modifying effects and vulnerable sub-populations.

**Results:**

We found that the case-severity rate of HFMD varied dramatically between cities, ranging from 0 to 8.09%. Cities with high case-severity rates were mainly clustered in Central China. By relating the case-severity rate to city-specific characteristics, we found that both the principal component characterized by a high level of social and economic development (RR = 0.823, 95%CI 0.739, 0.916) and another that characterized by warm and humid climate (RR = 0.771, 95%CI 0.619, 0.960) were negatively associated with the case-severity rate of HFMD. These estimations were consistent across age and gender sub-populations.

**Conclusion:**

Except for the type of infected pathogen, the case-severity rate of HFMD was closely related to city development and meteorological factor. These findings suggest that social and environmental factors may also play an important role in the progress of severe HFMD.

**Supplementary Information:**

The online version contains supplementary material available at 10.1186/s12199-020-00927-9.

## Introduction

Hand-foot-and-mouth disease (HFMD) is a contagious disease caused by the enterovirus. Most cases of HFMD are mild and self-limited, but a small proportion could further develop to severe complications affecting the central nervous systems [1, 2]. Due to its rapid progression and the lack of effective antiviral medications [3–5], the prognosis of severe HFMD cases is usually poor [6, 7]. Patients often suffer due to high medical expenses [8] and poor quality of life [9]. Enterovirus A71 (EV-A71) has been shown to be the dominant pathogen causing severe HFMD [1, 10]. Approximately 80% and 95% of severe and fatal cases test positive for EV-A71, respectively [1, 11]. However, among the EV-A71 infected cases, only 23.88% and 1.89% develop into severe and fatal cases [12], implying that there still remain other unidentified risk factors for severe HFMD.

The overwhelming majority of prior studies mainly focused on examining the associations between severe HFMD and individual-level risk factors, such as individual demographic characteristics, early clinical manifestations, and genetic susceptibility. It has been found that younger [13] and male sex [14] were more vulnerable to severe HFMD, while breastfeeding and hand washing after playing [15] were found to be protective. Clinical manifestations including high fever, vomiting, myoclonic twitching, and elevated serum interleukins and interferon-γ levels were found to be early indicators of severe cases [16]. The presence of the rs4290270 SNP in the TPH2 gene was associated with increased susceptibility to severe HFMD [17].

Although those above individual-level studies have provided important insights for identifying high-risk individuals from a clinical perspective, there is still an urgent need to clarify the associations between regional-level risk factors and severe HFMD. This would provide a deeper understanding of why severe HFMD cases are more likely to occur in specific regions, and then provide evidences on how to better control severe HFMD from a public health perspectives. To the best of our knowledge, rare studies have paid special attention to the associations between regional-level risk factors and severe HFMD [18–21]. Only a few studies sporadically reported higher population density [21], rural living [18], and lower socioeconomic status [18] would raise the epidemic of severe HFMD. In fact, there were remaining other regional-level factors should be considered when estimating their impact on epidemic of severe HFMD, such as medical resource allocation, meteorological features, GDP, and population scale. Notably, previous studies were mainly limited in a specific province, and a nationwide study covering multidimensional factors is still in lack.

Therefore, to address the above research gap, we conducted this nationwide study including 143 prefectural-level cities and abundant city-level characteristics to identify regional-level risk factors for the severe HFMD.

## Methods

### City selection

In the current study, the observational units were cities in mainland China. For each city, the city-specific characteristics and case-severity rate were used as the covariates and outcome, respectively. City selection was based on the availability of meteorological covariates and the case-severity rate. The case-severity rate was calculated for 293 cities in mainland China based on daily counts of HFMD clinical cases [22]. Meteorological covariates were collected from 646 national ground meteorological stations through the China Meteorological Data Sharing Service System. After matching meteorological stations with their corresponding cities, 143 cities were included in the current study [22, 23]. Each of those 143 cities was further defined as the main central urban area in each prefecture (i.e., the prefectural-level city) [22]. This is because prefecture-level cities usually have a larger population which is more important for infectious disease control. Additionally, when compared with small cities, the prefecture-level cities have better surveillance systems that can guarantee data quality.

### City-specific case-severity rate of HFMD

The city-specific case-severity rate was chosen as the outcome variable due to its representation of the regional disease burden of severe HFMD [1]. It was defined as the total number of severe cases divided by the total number of probable cases from 1 January 2008 to 30 December 2014. Severe cases were defined as clinical cases with any CNS complications, cardiopulmonary dysfunction, or both. Probable cases were defined as patients with a papular or vesicular rash on the hands, feet, mouth, or buttocks, with or without fever [1]. All cases were collected from the daily counts of clinical HFDM cases through the China Information System for Disease Control and Prevention. Using this system, other variables, including patient age, gender, enterovirus serotypes, time from symptom onset to diagnosis, and severity (mild or severe), were also extracted. In addition, as over 99% of HFMD cases occurred among children under the age of 12 years (i.e., children in elementary school and below) according to our preliminary analysis [22]. Therefore, in the current study, we mainly focused on the incidence of HFMD among children aged 0–12 years.

The starting point of the observation period was 1 January 2008, as all probable and laboratory-confirmed HFMD cases were required to be reported to the Chinese Centre for Disease Control and Prevention (China CDC) since 1 January 2008 [1], before when relevant data were not available. The endpoint was set at 30 December 2014 because the EV71 vaccine was approved for marketing on 3 December 2015 [24], after when the epidemiology of HFMD might has become altered.

### City-specific characteristics

The city-specific characteristics mainly included socioeconomic and meteorological variables. The socioeconomic variables were collected from the China City Statistical Yearbook [25] including demographic variables (population density and rate of population increase), economic variables (GDP per person and rate of GDP increase), health resources (number of licensed physicians, hospital beds, and hospitals per 1000 persons), traffic (total travel passengers per year), number of elementary school students per 1000 persons, and per capita public green areas. Meteorological variables, including relative humidity, temperature, rainfall, and sunshine hours, were extracted from daily meteorological monitoring data. Arithmetic means for meteorological variables were calculated for each 143 cities to exhibit the city level meteorological differences.

### Statistical analysis

#### Comparisons of city-specific characteristics between city groups

To answer why severe HFMD cases prone to occur at specific regions, two city groups were defined. Cities with a high case-severity and a low incidence were classified as severe HFMD burdened areas (ranked 51–100% in case-severity rate, and 1–50% in incidence). Cities with a low case-severity rate and a high incidence were classified as less burdened areas (ranked 1–50% in case-severity rate, and 51–100% in incidence). Then comparisons of city-specific characteristics between these two groups were conducted.

#### Dimensionality reduction of city-specific characteristics

To reduce multicollinearity, city-specific covariates with a variance inflation factor (VIF) ≥ 5 [26] were compressed by principal component analysis (PCA) [27] (see Additional file 1: Table S2 for the variance inflation factor (VIF) of each city-specific variable). New variables (principal components, PCs) and city-specific covariates with a VIF < 5 were used as regressors in the subsequently generated core model.

#### Estimating associations between case-severity rate and city-specific characteristics

A Poisson regression was applied to examine the associations between the case-severity rate and city-specific characteristics. The principal components and city-specific variables (VIF < 5) were the regressors, the cumulative number of severe cases during the observation period was the outcome, and the natural logarithm of the corresponding probable cases was the offset.

### Stratification analysis

Stratification analysis on gender and age-group were further carried out based on the core model. Gender and age were the most important demographic characteristics [1, 28] [29–34]; therefore, stratification analyses on gender and age were used to identify potential vulnerable sub-populations. The cutoff value of age group was 2 years old (age ≥ 2 years vs. age < 2 years), as 2 years has been observed to be the peak age of both incidence and severity-case rate [1, 28].

All statistical analyses were performed using R software (Version 3.6.1; R Core Team, 2019), mainly using the packages “GLM” and “principal.” Spatial distribution maps of HFMD incidence and case-severity rate were made using ArcGIS Pro (version 2.4, authorization number: EFL734321752).

## Results

### Spatial distribution of case-severity rate and incidence of HFMD

**Fig. 1:**
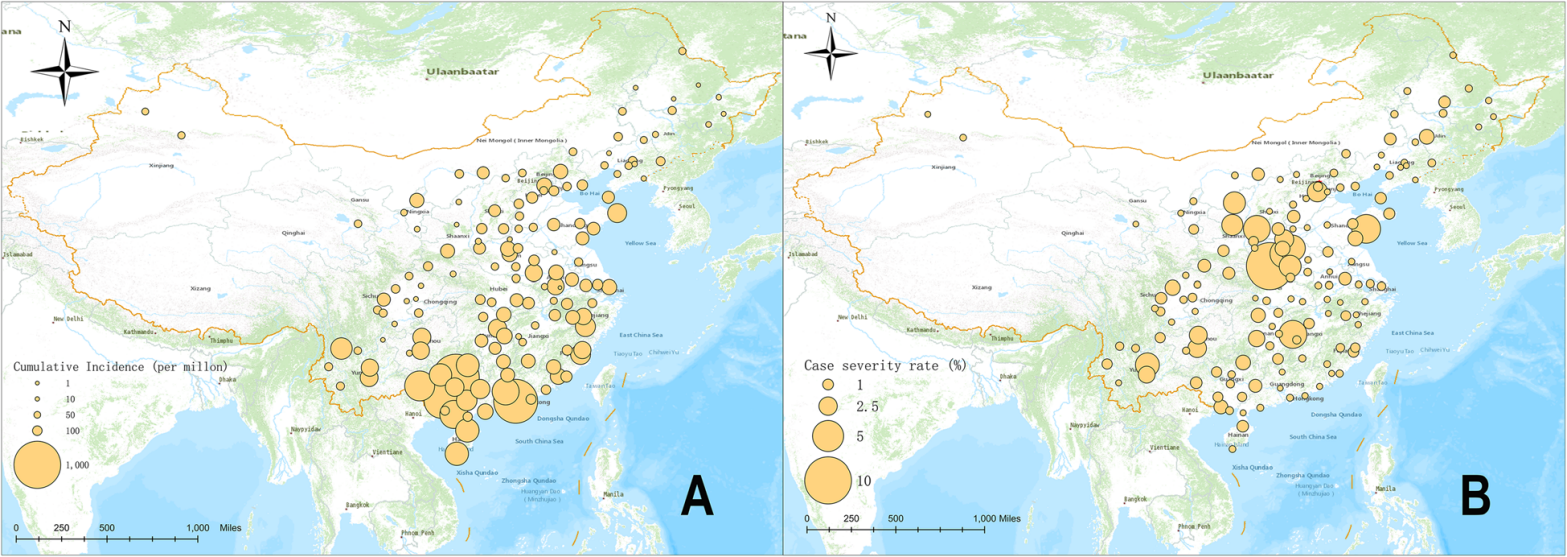
The spatial distribution of case-severity rate and HFMD incidence (circle diameter at each coordinate is proportional to the corresponding value). **a** Cumulative incidence of HFMD (per million). **b** Case-severity rate of severe HFMD (%)

In the current analysis, 143 cities were included, covering the majority of the medium and large size cites cities in mainland China. A total of 3,656,006 probable cases, including 27,690 severe cases (cases-severity risk 0.76%) were observed in the period from 2008 to 2014. The 7-year cumulative incidence of HFMD ranged from 11.70 per million to 744.97 per million, and the case-severity rate ranged from 0 to 8.09%. Discrepant spatial distributions of the case-severity rate and HFMD incidence were observed. High HFMD incidence was observed in the Pearl River Delta and the South Area (Fig. 1a), while high case-severity rates were observed was in Central China, including Henan, Shandong, and Shanxi provinces (Fig. 1b).

### Comparison of city-specific characteristics between severe HFMD burdened and less burdened areas

There were 30 cities classified into severe HFDM burdened areas, and 30 cities into the less burdened areas (see Additional file 1: Table S1 for classification of severe HFMD burdened and less burdened areas). Comparisons between those two areas revealed that severe HFMD burdened areas had lower GDP and GDP per person, lower population density, fewer licensed doctors, fewer per capita public green areas, lower average temperature, and less average rainfall (Table 1).

**Table 1:** Comparison of social demographic, economic, and meteorological qualities between subgroups

Variables	Less burdened areas (***N*** = 30)	Burdened areas (***N*** = 30)	***t/Z***	***P*** value
**Average population** (millions)	93.11(56.07, 230.74)	96.94(66.34, 142.34)	− 0.296	0.767
**Population increase rate** (%)	5.70(2.93, 7.75)	6.08(1.14, 13.32)	− 0.451	0.652
Population density (people/km2)	1184.40(444.56, 1629.07)	500.31(287.98, 893.66)	− 2.632	0.008
**Number of elementary school students** (10,000)	6.65(4.67, 16.96)	7.60(5.26, 11.71)	− 0.444	0.657
**GDP** (CNY)	4608857.00 (2483430.00, 21241202.80)	2659869.50 (1176241.00, 4537211.50)	− 2.750	0.006
**GDP per person** (CNY)	55099.00 (36597.25, 66345.25)	30374.00 (17131.75, 47843.25)	− 3.947	< 0.001
**GDP increase rate** (%)	14.39 ± 2.10	14.46 ± 3.38	− 0.092	0.927
**Traffic passengers** (10,000)	9904.45 (4101.32, 20577.51)	6844.86 (4567.86, 11211.64)	− 1.124	0.261
**Number of hospitals** (per thousand)	48.00(28.50, 110.50)	58.50(33.00, 94.25)	− 0.569	0.569
**Number of hospital beds** (per thousand)	7261.50(3342.00, 20019.00)	4756.00(3237.50, 7554.25)	− 1.434	0.152
**Number of licensed doctors** (per thousand)	3623.00(1684.75, 9729.25)	2198.50(1355.00, 3528.00)	− 1.959	0.050
**Per capita public green area** (m^2^)	36.00(28.25, 54.75)	25.00(14.75, 33.00)	− 3.491	< 0.001
**Average temperature** (°F)	176.96 ± 38.93	138.05 ± 51.39	3.306	0.002
**Average relative humidity** (%)	70.04 ± 8.63	65.85 ± 9.03	1.837	0.071
**Average rainfall** (mm)	35.89 ± 15.50	23.42 ± 11.72	3.514	0.001
**Average sunshine** (min)	50.81 ± 8.18	54.22 ± 15.08	− 1.089	0.282

### Associations between case-severity rate and city-specific characteristics

City development and meteorological indicators were two principal components generated by PCA. The city development indicator was characterized by high levels of social and economic development level and accounted for 66% of the total variance. The meteorological indicator was characterized by warm and humid climate and accounted for 34% of the total variance. The case-severity rate of HFMD was negatively associated with GDP increase rate (RR = 0.745, *P* = 0.009), city development indicator (RR = 0.823 *P* < 0.001), and meteorological indicator (RR = 0.771, *P* = 0.022) (Table 2).

**Table 2:** The Poisson regression model for associations between case-severity rate and city-specific characteristics

Variables	Estimate	SE	***t***	***P*** value	RR (95%CI) ^**b**^	RR (95%CI) ^**a**^
Population increase rate	0.104	0.175	0.595	0.553	1.109 (0.788, 1.562)	1.024 (0.718, 1.459)
Population density	− 0.026	0.100	− 0.261	0.795	0.974 (0.801, 1.186)	1.041 (0.820, 1.323)
GDP increase rate	− 0.294	0.112	− 2.635	**0.009**	0.745 (0.599, 0.927)	0.921 (0.777, 1.092)
Per capita public green areas	− 0.105	0.077	− 1.362	0.175	0.901 (0.775, 1.047)	0.868 (0.751, 1.003)
City development	− 0.195	0.055	− 3.561	**< 0.001**	0.823 (0.739, 0.916)	0.893 (0.821, 0.970)
Meteorological feature	− 0.261	0.112	− 2.321	**0.022**	0.771 (0.619, 0.960)	0.723 (0.592, 0.882)
Intercept	− 4.666	0.137	− 33.952	< 0.001	0.009 (0.007, 0.012)	–

^a^The crude RR for case-severity rate

^b^The adjusted RR in multivariate Poisson regression

### Stratification analysis by gender and age group

**Fig. 2:**
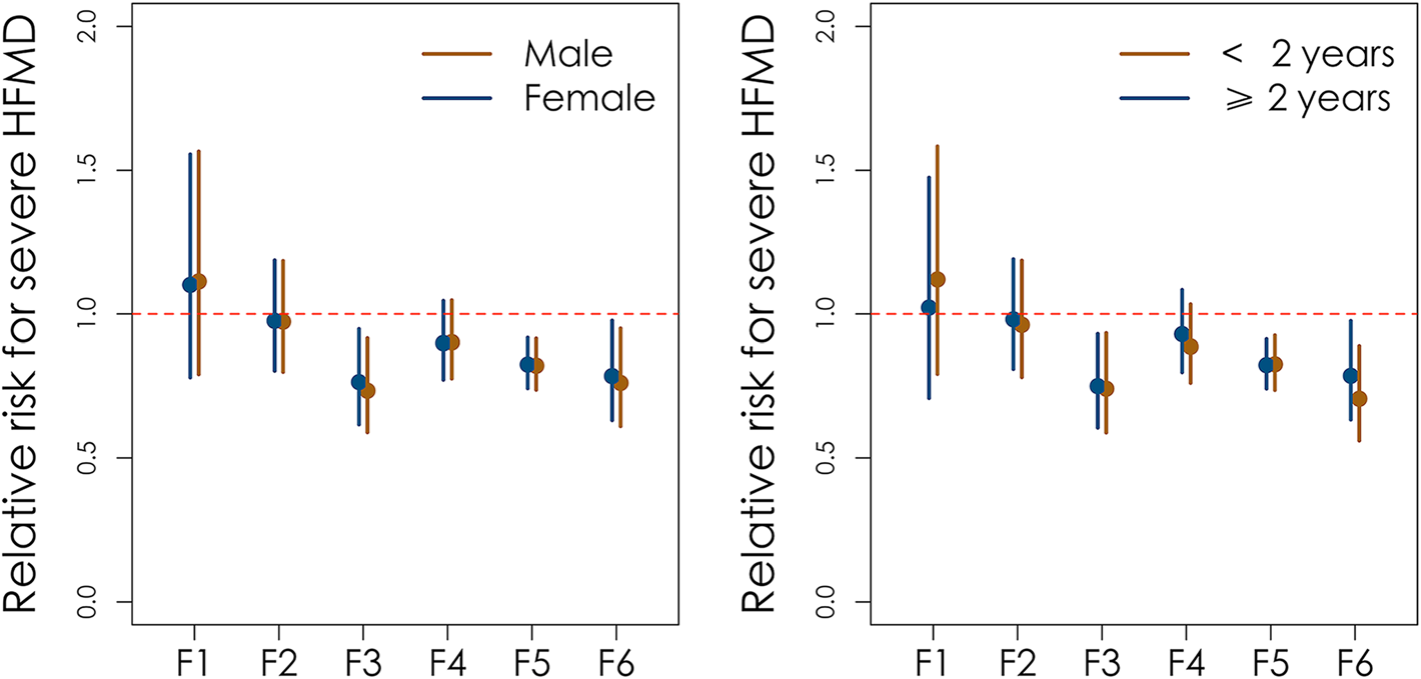
Stratified analysis by gender and age group. **a** Coefficients from the core model stratified by gender. **b** Coefficients from the core model stratified by age; F1, F2, F3, F4, F5, and F6 corresponded to the covariates (F1: population increase rate, F2: population density, F3: GDP increase rate, F4: per capita public green areas, F5: city development, F6: meteorological feature)

In the stratification analysis by gender, the results of the core model were consistent. Both in the male and female sub-populations, the GDP increase rate, city development indicator, and meteorological feature were all significantly negatively correlated with the case-severity rate (Table 3, Fig. 2a). When stratified by age, the results of the core model were also consistent. In the two age groups, the GDP increase rate, city development indicator, and meteorological feature were all significantly negatively correlated to the case-severity rate (Table 4, Fig. 2b).

**Table 3:** Stratified analysis of the Poisson regression model in male and female subgroups

Variables	Male		Female	
	RR ^**b**^	RR ^**a**^	RR ^**b**^	RR ^**a**^
Population increase rate	1.113 (0.791, 1.566)	1.012 (0.719, 1.441)	1.101 (0.780, 1.556)	1.041 (0.729, 1.487)
Population density	0.973 (0.799, 1.185)	1.040 (0.819, 1.323)	0.976 (0.803, 1.187)	1.041 (0.820, 1.323)
GDP increase rate	0.734 (0.589, 0.916)	0.912 (0.769, 1.083)	0.764 (0.616, 0.948)	0.937 (0.792, 1.108)
Per capita public green areas	0.902 (0.776, 1.048)	0.867 (0.751, 1.000)	0.899 (0.772, 1.046)	0.869 (0.750, 1.006)
City development	0.821 (0.737, 0.915)	0.897 (0.824, 0.975)	0.825 (0.742, 0.918)	0.886 (0.816, 0.963)
Meteorological feature	0.761 (0.610, 0.950)	0.715 (0.587, 0.872)	0.785 (0.631, 0.977)	0.734 (0.601, 0.897)
Intercept	0.010 (0.007, 0.013)	–	0.009 (0.007, 0.012)	–

^a^The crude RR for case-severity rate

^b^The adjusted RR in multivariate Poisson regression

**Table 4:** Stratified analysis of the Poisson regression model in different age groups

Variables	Greater than or equal to 2 years old		Less than 2 years old	
	RR ^**b**^	RR ^**a**^	RR ^**b**^	RR ^**a**^
Population increase rate	1.022 (0.708,1.475)	0.953 (0.659, 1.378)	1.120 (0.792, 1.584)	0.982 (0.681, 1.425)
Population density	0.981 (0.809,1.191)	1.045 (0.843, 1.305)	0.962 (0.781, 1.186)	1.041 (0.799, 1.357)
GDP increase rate	0.750 (0.605,0.931)	0.914 (0.779,1.073)	0.741 (0.588, 0.934)	0.897 (0.744, 1.080)
Per capita public green areas	0.930 (0.798,1.084)	0.894 (0.779, 1.027)	0.887 (0.761, 1.034)	0.839 (0.723, 0.973)
City development	0.823 (0.741,0.913)	0.906 (0.836, 0.981)	0.826 (0.736, 0.926)	0.894 (0.815, 0.981)
Meteorological feature	0.786 (0.633,0.976)	0.738 (0.612, 0.889)	0.706 (0.560, 0.890)	0.659 (0.534, 0.813)
Intercept	0.007 (0.005,0.009)	–	0.013 (0.01, 0.017)	–

^a^The crude RR for case-severity rate

^b^The adjusted RR in multivariate Poisson regression

## Discussion

Using nationwide data on severe HFMD, we observed the city-specific characteristics were related to regional differences in the case-severity rate. Our main findings suggest that beyond the type of infected pathogen, attention to social and environmental factors is necessary for controlling epidemics of severe HFMD. In addition, based on the spatial distribution of the case-severity rate and incidence, we found that areas of high HMFD incidence were mainly in the Pearl River Delta and South China, while areas of high case-severity rates were mainly located in Central China.

The city development indicator was negatively associated with the case-severity rate, indicating case-severity rate was higher in areas with lower economic development and insufficient medical resources. This phenomenon might be due to that the rural living [35, 36], lower socioeconomic status [21], and insufficient medical capacity [37] are more common in less developed areas. Poor personal hygiene, insufficient knowledge of disease, and delayed therapy increase the number of severe HFMD cases, thus resulting in a higher case-severity rate. Therefore, we suggest increasing the allocation of local medical resources and improving accessibility, as this might reduce the regional disease burden of severe HFMD.

We found that the meteorological feature indicator representing a warm and humid climate was negatively associated with the case-severity rate. One possible explanation might be that in a warm and humid climate, an increase of probable cases will be more pronounced than the increase of severe cases. At present, most of the researches support the idea that the number of probable cases would increase in a warm and humid climate [22, 38–40]. This is because higher temperatures may increase host activity, resulting in more frequent contact between infected and susceptible individuals. On humid days, enteroviruses can be easily attached to the small articles in the air, resulting in easier transmission of enteroviruses [40]. In addition, on sunny days, ultraviolet radiation could lead to inactivation of enteroviruses [39, 41]. However, the increase in severe cases would be less obvious than the increase in probable cases. This is because progression to severe HFMD not is only determined by enteroviral infection, but also depends on individual immunity [42] and medical treatment [33]. Therefore, in a warm and humid climate, a faster increase of total HFMD cases would accompanied by a decreased case-severity rate.

Previous studies have shown that both HFMD incidence [1] and case-severity rate [21] were positively correlated with the population density, while this relationship is non-significant in severe HFMD incidence [21]. In the current study, we found that the relationship between population density and the case-severity rate was non-significant. This might for the following reasons: first, we hypothesized that two opposing forces might drive the relationship between population density and case-severity rate. On the one hand, more densely populated areas typically have better socioeconomic status and sufficient medical resources. Children in those areas are often better cared by more educated caregivers (resulting in lower case-severity rate). On the other hand, more densely populated areas have a higher severe HFMD detection rate with more sufficient medical facilities (resulting in a higher case-severity rate). Therefore, given both of these trends, the relationship between population density and case-severity rate might be non-significant. Moreover, based on the interpretation of statistical modeling, the non-significance of the population density indicates its impact on the case-severity rate was weaker than the impact from the city development and meteorological indicators. Therefore, we suggest that in addition to physical isolation measures, adequate health resources and timely treatment are crucial for controlling severe HFMD.

This study had two major strengths. First, this is a city-level study. Unlike the individual-level studies focusing on the diagnosis and treatment of clinical cases, results from the current study are crucial for public health decision-making. Second, this is a nationwide study with multiple covariates, which can provide a stable estimation on the associations between case-severity rate and city-specific characteristics.

This study has four main limitations. The first one is the intrinsic nature of ecological study. Conclusions from the current study were based on the population scale, inference on other situations should be careful. The second limitation is related to the data quality. Since our data was collected from surveillance data, under-reporting from surveillance data might introduce extra basis. However, the cumulative numbers from 2008 to 2014 were used as outcome, which are more reliable than single observations under mild under-reporting conditions. The third limitation is that the city-specific EV-A71 infection rate was not included in the core model. This is because that laboratory-based diagnosis of HFMD was only available in a small fraction of the total HFMD cases. Therefore, city-specific EV-A71 infection rate were not available. The final limitation is the study period, which did not extend into most recent years. As the EV71 vaccine was approved for marketing on 3 December 2015 [24], the epidemiology of HFMD has since changed. Further studies comparing those associations before and after the introduction of the vaccine are necessary.

## Conclusion

In conclusion, the case-severity rate is an indicator of severe HFMD disease burden, unlike the incidence of HFMD, which is mainly affected by enterovirus, meteorological factors, and population density. The case-severity rate was closely related to medical resource allocation and the level of city development. Enhancing the medical resource allocation and improving it accessibility among rural population might result in improved disease prevention and control.

## Supplementary Information

**Additional file 1: Table S1** Classification of severe HFMD burdened and less burdened groups for the 143 cities. **Table S2** Variance inflation factor for city-specific characteristics. **Table S3** Principal component loadings on part of city-specific characteristics. **Figure S1** Visualization of the principal component analysis. A. The principal component loadings of the first three principal components. B. The map of variable-categorizing based on the first two principal components (PC_1, PC_2 correspond to the City development and Meteorological features, respectively.). **Table S4** The Poisson regression model for associations between city characteristics and case-severity rate in male cases. **Table S5** The Poisson regression model for associations between city characteristics and case-severity rate in female cases. **Table S6** The Poisson regression model for associations between city characteristics and case-severity rate in patients under two years. **Table S7** The Poisson regression model for associations between city characteristics and case-severity rate in patients above and equal to two years

## Data Availability

The meteorological data used in the study are available from the China Meteorological Data Network (http://data.cma.cn/en). The Socioeconomic data used in the study are available from the statistical yearbook (http://www.stats-sd.gov.cn/). Other data is available from the corresponding author on reasonable request.

## Abbreviations

CA-V16: Coxsackievirus A16
CDC: Center for Disease Control
EV-A71: Enterovirus 71
HFMD: Hand-foot-and-mouth disease
GDP: Gross domestic product
