# Supplementary material for: Regional-level risk factors for severe hand-foot-and-mouth disease: an ecological study from mainland China
Source: Environ Health Prev Med. 2021 Jan 8;26:4. doi: 10.1186/s12199-020-00927-9 (PMC7792012; doi:10.1186/s12199-020-00927-9)
Supplement: Supplementary file 1 — Additional file 1: Table S1 Classification of severe HFMD burdened and less burdened groups for the 143 cities. Table S2 Variance inflation factor for city-specific characteristics. Table S3 Principal component loadings on part of city-specific characteristics. Figure S1 Visualization of the principal component analysis. A. The principal component loadings of the first three principal components. B. The map of variable-categorizing based on the first two principal components (PC_1, PC_2 correspond to the City development and Meteorological features, respectively.). Table S4 The Poisson regression model for associations between city characteristics and case-severity rate in male cases. Table S5 The Poisson regression model for associations between city characteristics and case-severity rate in female cases. Table S6 The Poisson regression model for associations between city characteristics and case-severity rate in patients under two years. Table S7 The Poisson regression model for associations between city characteristics and case-severity rate in patients above and equal to two years [file 12199_2020_927_MOESM1_ESM.docx]

Supplementary Materials

Regional-level risk factors for severe hand-foot-and-mouth disease: an ecological study from mainland China

S1: Classification of severe HFMD burdened and less burdened areas

S2: The details of the principal component analysis

S3. Details of stratified analysis

**S1: Classification of the burdened and less burdened areas**

Cities with a high case-severity and a low incidence were classified as severe HFMD burdened areas (ranked 51 - 100% in case-severity rate, and 1 – 50% in incidence). Cities with a low case-severity rate and a high incidence were classified as less burdened areas (ranked 1 – 50% in case-severity rate, and 51 – 100% in incidence). Then comparisons of city-specific characteristics between these two groups were conducted. See table S1 for the classification of the 143 cities.

**Table S1** Classification of severe HFMD burdened and less burdened groups for the 143 cities

| **CityID** | **City** | **Case severity rate** | **Cumulative incidence** | **Rank of Case severity rate** | **Rank of cumulative incidence** | **City group** |
| --- | --- | --- | --- | --- | --- | --- |
| 1 | Yichang | 0.000 | 106.011 | Q1~Q2 | Q3~Q4 | Less Burdened area |
| 2 | Tianjing | 0.065 | 106.713 | Q1~Q2 | Q3~Q4 | Less Burdened area |
| 3 | Shanwei | 0.139 | 108.917 | Q1~Q2 | Q3~Q4 | Less Burdened area |
| 4 | Haozhou | 0.179 | 118.259 | Q1~Q2 | Q3~Q4 | Less Burdened area |
| 5 | Baoding | 0.139 | 128.932 | Q1~Q2 | Q3~Q4 | Less Burdened area |
| 6 | Xiamen | 0.330 | 129.071 | Q1~Q2 | Q3~Q4 | Less Burdened area |
| 7 | Huangshan | 0.018 | 130.282 | Q1~Q2 | Q3~Q4 | Less Burdened area |
| 8 | Jindezhen | 0.081 | 133.609 | Q1~Q2 | Q3~Q4 | Less Burdened area |
| 9 | Huangshi | 0.131 | 138.537 | Q1~Q2 | Q3~Q4 | Less Burdened area |
| 10 | Taiyuan | 0.045 | 140.171 | Q1~Q2 | Q3~Q4 | Less Burdened area |
| 11 | Sanmenxia | 0.217 | 141.781 | Q1~Q2 | Q3~Q4 | Less Burdened area |
| 12 | Changzhou | 0.245 | 145.614 | Q1~Q2 | Q3~Q4 | Less Burdened area |
| 13 | Jinan | 0.144 | 149.097 | Q1~Q2 | Q3~Q4 | Less Burdened area |
| 14 | Wuhan | 0.112 | 159.912 | Q1~Q2 | Q3~Q4 | Less Burdened area |
| 15 | Ganzhou | 0.318 | 174.961 | Q1~Q2 | Q3~Q4 | Less Burdened area |
| 16 | Shaoguan | 0.067 | 177.673 | Q1~Q2 | Q3~Q4 | Less Burdened area |
| 17 | Yinchuan | 0.274 | 180.213 | Q1~Q2 | Q3~Q4 | Less Burdened area |
| 18 | Longyan | 0.236 | 185.224 | Q1~Q2 | Q3~Q4 | Less Burdened area |
| 19 | Bengbu | 0.050 | 193.612 | Q1~Q2 | Q3~Q4 | Less Burdened area |
| 20 | Yangjiang | 0.274 | 205.887 | Q1~Q2 | Q3~Q4 | Less Burdened area |
| 21 | Zhuzhou | 0.313 | 206.526 | Q1~Q2 | Q3~Q4 | Less Burdened area |
| 22 | Zhanghzou | 0.077 | 211.483 | Q1~Q2 | Q3~Q4 | Less Burdened area |
| 23 | Jinhua | 0.068 | 220.882 | Q1~Q2 | Q3~Q4 | Less Burdened area |
| 24 | Hefei | 0.394 | 230.380 | Q1~Q2 | Q3~Q4 | Less Burdened area |
| 25 | Changsha | 0.143 | 250.000 | Q1~Q2 | Q3~Q4 | Less Burdened area |
| 26 | Lishui | 0.147 | 282.292 | Q1~Q2 | Q3~Q4 | Less Burdened area |
| 27 | Sanya | 0.252 | 338.627 | Q1~Q2 | Q3~Q4 | Less Burdened area |
| 28 | Guanzhou | 0.045 | 401.858 | Q1~Q2 | Q3~Q4 | Less Burdened area |
| 29 | Shenzhen | 0.259 | 696.319 | Q1~Q2 | Q3~Q4 | Less Burdened area |
| 30 | Liuzhou | 0.377 | 744.972 | Q1~Q2 | Q3~Q4 | Less Burdened area |
| 31 | Suihua | 1.173 | 13.278 | Q3~Q4 | Q1~Q2 | Burdened area |
| 32 | Yanan | 3.043 | 15.943 | Q3~Q4 | Q1~Q2 | Burdened area |
| 33 | Zhaotong | 0.895 | 16.257 | Q3~Q4 | Q1~Q2 | Burdened area |
| 34 | Nanchong | 0.606 | 17.928 | Q3~Q4 | Q1~Q2 | Burdened area |
| 35 | Xinxiang | 2.626 | 24.041 | Q3~Q4 | Q1~Q2 | Burdened area |
| 36 | Xuzhou | 0.908 | 25.105 | Q3~Q4 | Q1~Q2 | Burdened area |
| 37 | Yulin | 3.218 | 33.159 | Q3~Q4 | Q1~Q2 | Burdened area |
| 38 | Guyuan | 0.805 | 33.559 | Q3~Q4 | Q1~Q2 | Burdened area |
| 39 | Yichun | 4.401 | 35.181 | Q3~Q4 | Q1~Q2 | Burdened area |
| 40 | Ankang | 1.260 | 38.753 | Q3~Q4 | Q1~Q2 | Burdened area |
| 41 | Yuncheng | 0.812 | 39.256 | Q3~Q4 | Q1~Q2 | Burdened area |
| 42 | Changchun | 1.788 | 40.572 | Q3~Q4 | Q1~Q2 | Burdened area |
| 43 | Nanyang | 8.091 | 62.813 | Q3~Q4 | Q1~Q2 | Burdened area |
| 44 | Nanchang | 0.965 | 64.382 | Q3~Q4 | Q1~Q2 | Burdened area |
| 45 | Jian | 0.530 | 66.241 | Q3~Q4 | Q1~Q2 | Burdened area |
| 46 | Panzhihua | 0.453 | 67.009 | Q3~Q4 | Q1~Q2 | Burdened area |
| 47 | Chifeng | 0.567 | 69.752 | Q3~Q4 | Q1~Q2 | Burdened area |
| 48 | Langfang | 2.872 | 74.204 | Q3~Q4 | Q1~Q2 | Burdened area |
| 49 | Xinyang | 0.627 | 74.997 | Q3~Q4 | Q1~Q2 | Burdened area |
| 50 | Baoshan | 0.909 | 75.291 | Q3~Q4 | Q1~Q2 | Burdened area |
| 51 | Leshan | 0.665 | 75.804 | Q3~Q4 | Q1~Q2 | Burdened area |
| 52 | Haerbin | 0.437 | 77.862 | Q3~Q4 | Q1~Q2 | Burdened area |
| 53 | Zhumadian | 3.207 | 78.892 | Q3~Q4 | Q1~Q2 | Burdened area |
| 54 | Jingtai | 1.358 | 79.741 | Q3~Q4 | Q1~Q2 | Burdened area |
| 55 | Tongliao | 1.192 | 83.373 | Q3~Q4 | Q1~Q2 | Burdened area |
| 56 | Mianyang | 1.861 | 84.401 | Q3~Q4 | Q1~Q2 | Burdened area |
| 57 | Qinzhou | 1.622 | 91.044 | Q3~Q4 | Q1~Q2 | Burdened area |
| 58 | Tonghua | 0.439 | 91.349 | Q3~Q4 | Q1~Q2 | Burdened area |
| 59 | Hanzhong | 1.418 | 94.511 | Q3~Q4 | Q1~Q2 | Burdened area |
| 60 | Changde | 1.688 | 95.464 | Q3~Q4 | Q1~Q2 | Burdened area |
| 61 | Yichun | 0.317 | 11.700 | Q1~Q2 | Q1~Q2 | - |
| 62 | Qiqihaer | 0.270 | 18.286 | Q1~Q2 | Q1~Q2 | - |
| 63 | Fuxin | 0.066 | 19.463 | Q1~Q2 | Q1~Q2 | - |
| 64 | Jixi | 0.057 | 20.034 | Q1~Q2 | Q1~Q2 | - |
| 65 | Jiamusi | 0.150 | 24.514 | Q1~Q2 | Q1~Q2 | - |
| 66 | Dandong | 0.000 | 27.003 | Q1~Q2 | Q1~Q2 | - |
| 67 | Suining | 0.337 | 27.606 | Q1~Q2 | Q1~Q2 | - |
| 68 | Bazhong | 0.251 | 28.349 | Q1~Q2 | Q1~Q2 | - |
| 69 | Yibin | 0.124 | 30.089 | Q1~Q2 | Q1~Q2 | - |
| 70 | Benxi | 0.064 | 32.665 | Q1~Q2 | Q1~Q2 | - |
| 71 | Mudanjiang | 0.068 | 34.786 | Q1~Q2 | Q1~Q2 | - |
| 72 | Anshun | 0.328 | 39.042 | Q1~Q2 | Q1~Q2 | - |
| 73 | Liuan | 0.231 | 46.594 | Q1~Q2 | Q1~Q2 | - |
| 74 | Zhongwei | 0.104 | 48.617 | Q1~Q2 | Q1~Q2 | - |
| 75 | Kelamayi | 0.049 | 53.110 | Q1~Q2 | Q1~Q2 | - |
| 76 | Yinkou | 0.378 | 55.882 | Q1~Q2 | Q1~Q2 | - |
| 77 | Siping | 0.171 | 57.457 | Q1~Q2 | Q1~Q2 | - |
| 78 | Anqing | 0.138 | 58.908 | Q1~Q2 | Q1~Q2 | - |
| 79 | Heihe | 0.174 | 59.948 | Q1~Q2 | Q1~Q2 | - |
| 80 | Wulumuqi | 0.217 | 61.264 | Q1~Q2 | Q1~Q2 | - |
| 81 | Jinzhou | 0.017 | 62.886 | Q1~Q2 | Q1~Q2 | - |
| 82 | Datong | 0.081 | 63.599 | Q1~Q2 | Q1~Q2 | - |
| 83 | Anyang | 0.115 | 64.056 | Q1~Q2 | Q1~Q2 | - |
| 84 | Anshan | 0.032 | 64.617 | Q1~Q2 | Q1~Q2 | - |
| 85 | Xining | 0.026 | 66.360 | Q1~Q2 | Q1~Q2 | - |
| 86 | Zhangjiakou | 0.081 | 68.419 | Q1~Q2 | Q1~Q2 | - |
| 87 | Baicheng | 0.343 | 68.734 | Q1~Q2 | Q1~Q2 | - |
| 88 | Guangyuan | 0.153 | 70.591 | Q1~Q2 | Q1~Q2 | - |
| 89 | Linchang | 0.082 | 76.894 | Q1~Q2 | Q1~Q2 | - |
| 90 | Yaan | 0.221 | 77.588 | Q1~Q2 | Q1~Q2 | - |
| 91 | Chaoyang | 0.065 | 78.851 | Q1~Q2 | Q1~Q2 | - |
| 92 | Huaian | 0.158 | 80.071 | Q1~Q2 | Q1~Q2 | - |
| 93 | Chongqing | 0.106 | 82.106 | Q1~Q2 | Q1~Q2 | - |
| 94 | Tangshan | 0.363 | 82.386 | Q1~Q2 | Q1~Q2 | - |
| 95 | Baotou | 0.226 | 84.274 | Q1~Q2 | Q1~Q2 | - |
| 96 | Shenyang | 0.213 | 84.861 | Q1~Q2 | Q1~Q2 | - |
| 97 | Jingzhou | 0.403 | 86.534 | Q1~Q2 | Q1~Q2 | - |
| 98 | Hanghzou | 0.146 | 90.274 | Q1~Q2 | Q1~Q2 | - |
| 99 | Zhanjiang | 0.077 | 95.825 | Q1~Q2 | Q1~Q2 | - |
| 100 | Shantou | 0.105 | 99.400 | Q1~Q2 | Q1~Q2 | - |
| 101 | Shijiangzhua | 0.286 | 100.727 | Q1~Q2 | Q1~Q2 | - |
| 102 | Kaifeng | 4.028 | 102.707 | Q3~Q4 | Q3~Q4 | - |
| 103 | Nanping | 0.590 | 109.616 | Q3~Q4 | Q3~Q4 | - |
| 104 | Weifang | 0.951 | 112.230 | Q3~Q4 | Q3~Q4 | - |
| 105 | Changzhi | 1.360 | 112.717 | Q3~Q4 | Q3~Q4 | - |
| 106 | Nantong | 0.477 | 114.012 | Q3~Q4 | Q3~Q4 | - |
| 107 | Linfen | 4.173 | 115.736 | Q3~Q4 | Q3~Q4 | - |
| 108 | Qinghuangdao | 0.515 | 117.112 | Q3~Q4 | Q3~Q4 | - |
| 109 | Huhehaote | 1.324 | 143.942 | Q3~Q4 | Q3~Q4 | - |
| 110 | Hengyang | 0.498 | 144.404 | Q3~Q4 | Q3~Q4 | - |
| 111 | Dalian | 1.128 | 146.860 | Q3~Q4 | Q3~Q4 | - |
| 112 | Shaoyang | 2.109 | 154.901 | Q3~Q4 | Q3~Q4 | - |
| 113 | Qingdao | 4.554 | 155.592 | Q3~Q4 | Q3~Q4 | - |
| 114 | Rizhou | 1.819 | 159.533 | Q3~Q4 | Q3~Q4 | - |
| 115 | Chengdu | 1.169 | 162.131 | Q3~Q4 | Q3~Q4 | - |
| 116 | Nanjing | 1.334 | 162.310 | Q3~Q4 | Q3~Q4 | - |
| 117 | Binzhou | 1.019 | 175.668 | Q3~Q4 | Q3~Q4 | - |
| 118 | Xian | 1.118 | 176.050 | Q3~Q4 | Q3~Q4 | - |
| 119 | Quzhou | 0.833 | 177.644 | Q3~Q4 | Q3~Q4 | - |
| 120 | Chengde | 0.422 | 184.739 | Q3~Q4 | Q3~Q4 | - |
| 121 | Yueyang | 0.658 | 187.024 | Q3~Q4 | Q3~Q4 | - |
| 122 | shanghai | 0.545 | 192.656 | Q3~Q4 | Q3~Q4 | - |
| 123 | Zhenzhou | 1.839 | 192.725 | Q3~Q4 | Q3~Q4 | - |
| 124 | Beijing | 0.726 | 195.386 | Q3~Q4 | Q3~Q4 | - |
| 125 | Xuchang | 4.466 | 198.450 | Q3~Q4 | Q3~Q4 | - |
| 126 | Fuyang | 0.416 | 231.770 | Q3~Q4 | Q3~Q4 | - |
| 127 | Ningde | 0.743 | 233.333 | Q3~Q4 | Q3~Q4 | - |
| 128 | Kunming | 3.593 | 233.396 | Q3~Q4 | Q3~Q4 | - |
| 129 | Guiyang | 2.538 | 238.084 | Q3~Q4 | Q3~Q4 | - |
| 130 | Yuxi | 2.209 | 245.380 | Q3~Q4 | Q3~Q4 | - |
| 131 | Zunyi | 2.406 | 247.132 | Q3~Q4 | Q3~Q4 | - |
| 132 | Laibin | 0.514 | 255.821 | Q3~Q4 | Q3~Q4 | - |
| 133 | Weihai | 1.083 | 264.183 | Q3~Q4 | Q3~Q4 | - |
| 134 | Wuzhou | 1.448 | 268.761 | Q3~Q4 | Q3~Q4 | - |
| 135 | Fuzhou | 0.975 | 280.554 | Q3~Q4 | Q3~Q4 | - |
| 136 | Yulin | 0.704 | 292.434 | Q3~Q4 | Q3~Q4 | - |
| 137 | Lijiang | 0.532 | 307.461 | Q3~Q4 | Q3~Q4 | - |
| 138 | Chizhou | 0.919 | 322.582 | Q3~Q4 | Q3~Q4 | - |
| 139 | Guizhou | 1.796 | 329.963 | Q3~Q4 | Q3~Q4 | - |
| 140 | Haikou | 1.169 | 340.875 | Q3~Q4 | Q3~Q4 | - |
| 141 | Beihai | 0.432 | 421.123 | Q3~Q4 | Q3~Q4 | - |
| 142 | Baise | 1.194 | 454.512 | Q3~Q4 | Q3~Q4 | - |
| 143 | Nanning | 0.857 | 554.733 | Q3~Q4 | Q3~Q4 | - |

**S2. The details of the principal component analysis**

Original city-specific variables with variance inflation factor (VIF) greater than or equal to five were included in the principal component analysis (PCA) for dimensionality reduction [1]. Those original variables contained average population; number of pupils; GDP; passenger traffic volume; licensed physicians, beds, and hospitals per 1000 persons; relative humidity, temperature, rainfall, and sunshine hours (Table S2).

Two principal components were generated and jointly explained almost all the total variance (100%), of which the first principal component accounts for 66%, and the second principal component for 34% of the total variance, respectively. The first principal component was city development indicator as it mainly correlated with average population (loading=0.95), number of elementary school students (loading=0.92), GDP (loading=0.94), traffic passengers (loading=0.78), GDP per person (loading=0.52), number of hospitals (loading=0.87), number of beds(loading=0.96), and number of licensed doctors (loading=0.97). Higher value in city development indicator indicated better city development level. The second principal component was meteorological indicator as it mainly correlated with average temperature (loading=0.85), average relative humidity (loading=0.90), average rainfall (loading=0.88), and average sunshine (loading=-0.85). Cities with higher meteorological indicator were characterized by warm and humid and sunny climate (Table S3, Fig. S1).

**Table S2** Variance inflation factor for city-specific characteristics

| NO | Variables | VIF | Included in PCA † |
| --- | --- | --- | --- |
| 1 | Average population | 34.1 | Included |
| 2 | Population increase rate | 2.2 | Excluded |
| 3 | Population density | 2.2 | Excluded |
| 4 | No. of elementary school students | 18.8 | Included |
| 5 | GDP † | 25.3 | Included |
| 6 | GDP per person | 5.0 | Included |
| 7 | GDP increase rate | 1.9 | Excluded |
| 8 | Traffic passengers | 5.6 | Included |
| 9 | Number of hospitals | 7.3 | Included |
| 10 | Number of beds | 37.9 | Included |
| 11 | Number of licensed doctors | 29.0 | Included |
| 12 | Per capita public green areas | 3.8 | Excluded |
| 13 | Average temperature | 4930.2 | Included |
| 14 | Average relative humidity | 75.3 | Included |
| 15 | Average rainfall | 9.0 | Included |
| 16 | Average sunshine | 8.6 | Included |

† : Variables with variance inflation factor (VIF) greater than or equal to 5 were included in the principal component analysis (PCA) [1].

**Table S3** Principal component loadings on part of city-specific characteristics

| Variables | RC1 | RC2 | h2 | u2 | com |
| --- | --- | --- | --- | --- | --- |
| Average population | 0.95 | 0.03 | 0.90 | 0.10 | 1.00 |
| No. of elementary school students | 0.92 | 0.14 | 0.87 | 0.13 | 1.00 |
| GDP | 0.94 | 0.01 | 0.88 | 0.12 | 1.00 |
| Traffic passengers | 0.78 | 0.19 | 0.64 | 0.36 | 1.10 |
| GDP per person | 0.52 | -0.15 | 0.29 | 0.71 | 1.20 |
| Number of hospitals | 0.87 | -0.05 | 0.77 | 0.23 | 1.00 |
| Number of beds | 0.96 | -0.03 | 0.93 | 0.07 | 1.00 |
| Number of licensed doctors | 0.97 | -0.03 | 0.93 | 0.07 | 1.00 |
| Average temperature | 0.07 | 0.85 | 0.72 | 0.28 | 1.00 |
| Average relative humidity | -0.05 | 0.90 | 0.81 | 0.19 | 1.00 |
| Average rainfall | -0.03 | 0.88 | 0.78 | 0.22 | 1.00 |
| Average sunshine | -0.05 | -0.85 | 0.73 | 0.27 | 1.00 |


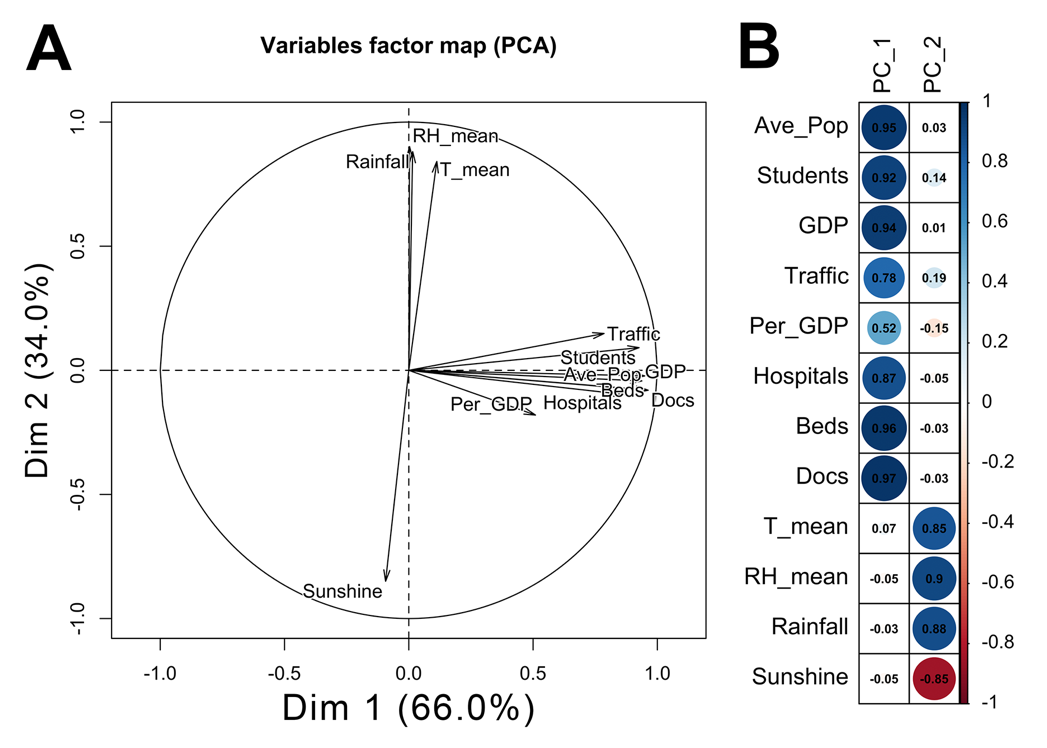


**Fig. S1** Visualization of the principal component analysis

A. The principal component loadings of the first three principal components

B. The map of variable-categorizing based on the first two principal components (PC_1, PC_2 correspond to the City development and Meteorological features, respectively.)

**S3. Details of stratification analysis**

**Stratification analysis within male subpopulation**

Similar results were generated from the male subgroup. The case-severity risk was significantly negatively correlated with GDP increase rate (RR = 0.734, *P* = 0.007), city development indicator (RR=0.821 *P* = 0.001), and meteorological indicator (RR = 0.761, *P* = 0.017) (Table S4).

**Table S4** The Poisson regression model for associations between city characteristics and case-severity rate in male cases

| Variables | Estimate | SE | *t* | *P*-value | RR | 95% CI for RR |
| --- | --- | --- | --- | --- | --- | --- |
| Population increase rate | 0.107 | 0.174 | 0.616 | 0.539 | 1.113 | 0.791~1.566 |
| Population density | -0.027 | 0.101 | -0.273 | 0.785 | 0.973 | 0.799~1.185 |
| GDP increase rate | -0.309 | 0.113 | -2.740 | 0.007 | 0.734 | 0.589~0.916 |
| Per capita public green areas | -0.103 | 0.077 | -1.344 | 0.181 | 0.902 | 0.776~1.048 |
| City development | -0.197 | 0.055 | -3.567 | 0.001 | 0.821 | 0.737~0.915 |
| Meteorological features | -0.272 | 0.113 | -2.416 | 0.017 | 0.761 | 0.610~0.950 |
| Intercept | -4.632 | 0.138 | -33.646 | <0.001 | 0.010 | 0.007~ 0.013 |

**Stratified analysis within female subpopulation**

Similar results were generated from the female subgroup. The case-severity risk was significantly negatively correlated with GDP increase rate (RR = 0.764, *P* = 0.016), city development indicator (RR=0.825 *P* = 0.001), and meteorological indicator (RR = 0.785, *P* = 0.032) (Table S5).

**Table S5** The Poisson regression model for associations between city characteristics and case-severity rate in female cases

| Variables | Estimate | SE | *t* | *P*-value | RR | 95% CI for RR |
| --- | --- | --- | --- | --- | --- | --- |
| Population increase rate | 0.097 | 0.176 | 0.548 | 0.584 | 1.101 | 0.780~1.556 |
| Population density | -0.024 | 0.100 | -0.240 | 0.811 | 0.976 | 0.803~1.187 |
| GDP increase rate | -0.269 | 0.110 | -2.441 | 0.016 | 0.764 | 0.616~0.948 |
| Per capita public green areas | -0.107 | 0.077 | -1.382 | 0.169 | 0.899 | 0.772~1.046 |
| City development | -0.192 | 0.054 | -3.545 | 0.001 | 0.825 | 0.742~0.918 |
| Meteorological features | -0.242 | 0.112 | -2.170 | 0.032 | 0.785 | 0.631~0.977 |
| Intercept | -4.722 | 0.138 | -34.332 | <0.001 | 0.009 | 0.007~0.012 |

**Stratified analysis within individuals under 2 years**

Similar results were generated from the subgroup of cases under 2 years. The case-severity risk was significantly negatively correlated with GDP increase rate (RR= 0.741, *P* = 0.012), city development indicator (RR=0.826, *P* = 0.001), and meteorological indicator (RR = 0.706, *P* = 0.004) (Table S6).

**Table S6** The Poisson regression model for associations between city characteristics and case-severity rate in patients under two years

| Variables | Estimate | SE | *t* | *P*-value | RR | 95% CI for RR |
| --- | --- | --- | --- | --- | --- | --- |
| Population increase rate | 0.113 | 0.177 | 0.641 | 0.523 | 1.120 | 0.792~1.584 |
| Population density | -0.038 | 0.107 | -0.360 | 0.719 | 0.962 | 0.781~1.186 |
| GDP increase rate | -0.299 | 0.118 | -2.540 | 0.012 | 0.741 | 0.588~0.934 |
| Per capita public green areas | -0.120 | 0.078 | -1.534 | 0.127 | 0.887 | 0.761~1.034 |
| City development | -0.191 | 0.059 | -3.268 | 0.001 | 0.826 | 0.736~0.926 |
| Meteorological features | -0.349 | 0.118 | -2.944 | 0.004 | 0.706 | 0.560~0.890 |
| Intercept | -4.332 | 0.140 | -30.918 | <0.001 | 0.013 | 0.010~0.017 |

**Stratified analysis within individuals over and equal to 2 years**

Similar results were generated from the subgroup of cases over and equal to 2 years. The case-severity risk was significantly negatively correlated with GDP increase rate (RR = 0.750, *P* = 0.010), city development indicator (RR=0.823, *P* < 0.001), and meteorological indicator (RR = 0.786, *P* = 0.031) (Table S7).

**Table S7** The Poisson regression model for associations between city characteristics and case-severity rate in patients above and equal to two years

| Variables | Estimate | SE | *t* | *P*-value | RR | 95% CI for RR |
| --- | --- | --- | --- | --- | --- | --- |
| Population increase rate | 0.022 | 0.187 | 0.116 | 0.908 | 1.022 | 0.708~1.475 |
| Population density | -0.019 | 0.099 | -0.189 | 0.850 | 0.981 | 0.809~1.191 |
| GDP increase rate | -0.287 | 0.110 | -2.612 | 0.010 | 0.750 | 0.605~0.931 |
| Per capita public green areas | -0.072 | 0.078 | -0.924 | 0.357 | 0.930 | 0.798~1.084 |
| City development | -0.195 | 0.053 | -3.668 | <0.001 | 0.823 | 0.741~0.913 |
| Meteorological features | -0.241 | 0.111 | -2.178 | 0.031 | 0.786 | 0.633~0.976 |
| Intercept | -4.943 | 0.142 | -34.814 | <0.001 | 0.007 | 0.005~0.009 |

**Reference**

1. Akinwande MO, Dikko HG, Samson A. Variance Inflation Factor: As a Condition for the Inclusion of Suppressor Variable(s) in Regression Analysis. Open Journal of Statistics. 2015;Vol.05No.07:14.
